# 1357. Collateral consequence of COVID pandemic: Increased incidence of cat scratch disease.

**DOI:** 10.1093/ofid/ofac492.1186

**Published:** 2022-12-15

**Authors:** Maria L Praino, Peditric Infectologist, Grigioni Julia, Peditric Infectologist, Soledad Tineo, Peditric Infectologist, Mayra Martinez, Ana Caratozzolo, Analía Toledano, Claudia Cazes, Peditric Infectologist, María M Contrini, Eduardo L López

**Affiliations:** Hospital de Niños Ricardo Gutierrez, Ciudad Autonoma de Buenos Aires, Ciudad Autonoma de Buenos Aires, Argentina; Hospital de Niños Ricardo Gutierrez, Ciudad Autonoma de Buenos Aires, Ciudad Autonoma de Buenos Aires, Argentina; Hospital de Niños Ricardo Gutierrez, Ciudad Autonoma de Buenos Aires, Ciudad Autonoma de Buenos Aires, Argentina; Hospital de Niños Ricardo Gutierrez, Ciudad Autonoma de Buenos Aires, Ciudad Autonoma de Buenos Aires, Argentina; Pediatric Infectious Disease Program, Hospital de Niños Ricardo Gutiérrez, Universidad de Buenos Aires, Buenos Aires, Ciudad Autonoma de Buenos Aires, Argentina; Pediatric Infectious Diseases Program, Hospital de Niños "Dr. Ricardo Gutiérrez", Universidad de Buenos Aires, Buenos Aires, Ciudad Autonoma de Buenos Aires, Argentina; Hospital de Niños Ricardo Gutierrez, Ciudad Autonoma de Buenos Aires, Ciudad Autonoma de Buenos Aires, Argentina; Pediatric Infectious Disease Program, Hospital de Niños Ricardo Gutiérrez, Universidad de Buenos Aires, Buenos Aires, Ciudad Autonoma de Buenos Aires, Argentina; Pediatric Infectious Disease Program, Hospital de Niños Ricardo Gutiérrez, Universidad de Buenos Aires, Buenos Aires, Ciudad Autonoma de Buenos Aires, Argentina

## Abstract

**Background:**

Cat scratch disease (CSD) is a zoonosis caused primarily by *Bartonella henselae.*

The aim of our study was to determine the incidence CSD before the preventive and obligatory social isolation/distancing that was indicated in Argentina as a consequence of the SARS-CoV-2 pandemic on March 19, 2020 ("first period") and to compare it with the incidence since then to present ("second period").

**Methods:**

Retrospective analysis of patients with CSD admitted to the Infectious Diseases outpatient office at Ricardo Gutiérrez Children’s Hospital (a tertiary care center) from March 2012 to March 2022. Charts were reviewed for demographic and epidemiological data, duration of symptoms, clinical manifestations and systemic compromise.

**Results:**

Two hundred twenty-nine patients were observed during the study period. The median delay between symptoms onset and medical consultation was 15 days (IQR 9-30d), without statistically significant differences between periods The median age at presentation was 101.67 months (range 11.3 to 211.87 months) Age and gender were similar between periods.

The average number of cases per month was 1.63 (158 in 97 months) versus 3.23 (71 cases in 22 months) in the first and second periods, respectively, with an increasing incidence of 98%. The frequency of systemic compromise was higher in the second period: 47.89% versus 32.28% (p= 0.01).

The months with the highest incidence were February to July.

**Conclusion:**

A significant increase of CSD were observed during the COVID pandemic probably related to a closer children´s contact with pets due to the strict quarantine that health authorities implemented. Moreover, an unexpected finding was a higher proportion of patients with systemic CSD during the pandemic period.

**Disclosures:**

**All Authors**: No reported disclosures.

